# Ring finger protein 180 suppresses cell proliferation and energy metabolism of non-small cell lung cancer through downregulating C-myc

**DOI:** 10.1186/s12957-022-02599-x

**Published:** 2022-05-21

**Authors:** Yi Ding, Yi Lu, Xinjie Xie, Lei Cao, Shiying Zheng

**Affiliations:** 1 Department of Thoracic Surgery, The First Affiliated Hospital of Soochow University, NO. 188, Shizi Street, Suzhou, 215006 People’s Republic of China; 2 Department of Thoracic Surgery, Shanghai Pudong New Area People’s Hospital, Shanghai University of Medicine and Health Sciences, Shanghai, 201318 People’s Republic of China; 3 Department of Pathology, Shanghai Pudong New Area People’s Hospital, Shanghai University of Medicine and Health Sciences, Shanghai, 201318 People’s Republic of China

**Keywords:** Non-small cell lung cancer, RING finger protein 180, C-myc, Cell proliferation, Glycolysis, Ubiquitination

## Abstract

**Background:**

Non-small cell lung cancer (NSCLC) causes numerous deaths worldwide. however, biomarkers for NSCLC prognosis are scarce for its heterogeneity. Proteins containing the RING finger domain RING finger protein 180 (RNF180) is a key mediator for ubiquitination, which controls cell cycle and regulates progression in certain human tumors. However, the detailed function of RNF180 in NSCLC remains unclear. In the present study, we aimed to investigate the role of RNF180 and its molecule network in NSCLC.

**Methods:**

Quantitative real-time polymerase chain reaction and immunohistochemical staining were used to analyze RNF180 levels. RNA interference and lentiviral-mediated vector transfections were performed to silence and overexpress RNF180 in NSCLC cells. Furthermore, Cell Counting Kit-8 was used for assessing biological function of RNF180 in cell proliferation and a xenograft model for examining its function in vivo. The activity of glycolysis was determined by examining the level of the extracellular acidification rate (ECAR).

**Results:**

RNF180 expression decreased in NSCLC tissues, and its expression was positively correlated with the survival rate of patients with NSCLC. Moreover, RNF180 overexpression suppressed the proliferation and glycolytic activities in NSCLC cells and restricted its tumorigenicity in vivo. Furthermore, RNF180 silencing promoted the proliferation and glycolysis metabolism of NSCLC cells, whereas C-myc inhibitor disrupted these effects. The underlying anti-oncogene of RNF180 involved in C-myc downregulation via ubiquitin-dependent degradation.

**Conclusions:**

Together, these results firstly indicated the anti-tumor properties of RNF180 and its correlation with NSCLC progression, thereby endorsing the potential role of RNF180 as an efficient prognostic biomarker for tumor recurrence.

**Supplementary Information:**

The online version contains supplementary material available at 10.1186/s12957-022-02599-x.

## Background

Lung cancer has an extremely poor prognosis and high mortality that exceeds the combined mortality caused by three of the most common human cancers, including breast, colorectal, and pancreatic cancers [1]. The 5-year survival rate of lung cancer is <18%, and the rate of deaths occurring within 1 year after lung cancer diagnosis and treatment is over 50% [2]. Non-small cell lung cancer (NSCLC) accounts for approximately 85% of all lung cancers, and therapies for NSCLC primarily comprise radiotherapy, chemotherapy, and immunotherapy, depending on the cancer stage [1]. However, tumor resistance toward these therapies remains a key factor with regard to recurrence and further metastasis, leading to a worse prognosis [3, 4]. Therefore, gaining a deep insight into the molecule mechanism of NSCLC is critical for developing novel therapy (Table 1).

**Table 1:** Association of RNF180 expression with clinical characteristics of 93 patients with non-small cell lung cancer

Characteristics	Patients (***n***=93)	RNF180 mRNA level		***P***
		High (***n***=53)	Low (***n***=40)	
**Age**				ns
<60	51 (54.8)	21 (41.2)	30 (58.8)	
>60	42 (45.2)	32 (76.2)	10 (23.8)	
**Gender**				ns
Male	49 (52.7)	22 (44.8)	27 (55.2)	
Female	44 (47.3)	31 (70.5)	13 (29.5)	
**Tumor size (cm)**				<0.001
<4	48 (51.6)	31 (64.6)	17 (35.4)	
>4	45 (48.4)	22 (48.9)	23 (51.1)	
**Smoking status**				ns
Never	60 (64.5)	30 (50)	30 (50)	
Former and current smokers	33 (35.5)	23 (69.7)	10 (30.3)	
**Tumor grade**				ns
Well and moderately differentiated	72 (77.4)	34 (47.2)	38 (52.8)	
Poorly differentiated	21 (22.6)	19 (33.3)	2 (44.9)	
**Lymph node metastasis**				<0.001
Positive	46 (49.5)	25 (54.3)	21 (45.7)	
Negative	47 (50.5)	28 (59.6)	19 (40.4)	
**Disease stage**				<0.05
I	28 (30.1)	18 (64.3)	10 (35.7)	
II	33 (35.4)	19 (57.6)	14 (42.4)	
III	32 (34.5)	16 (50)	16 (50)	

Differences between groups were done by the chi-square test

Biomarker tests can distinguish the prognosis of patients with NSCLC at certain clinical stages [5]. Therefore, it is crucial to recognize and develop practical molecular markers for predicting tumor recurrence and progression. Indeed, the enormous metabolic demands for tumor cell proliferation results in the physiological switching from mitochondrial function to aerobic glycolysis; this switching enables the tumor cells to acquire essential components from the generated byproducts, including nucleotides for DNA replication, amino acids for protein synthesis, and lipids for cell membrane formation [6, 7]. The metabolism of cancer cells are effectively different from that of normal cells, which are mainly dependent on aerobic glycolysis process of generating adenosine triphosphate (ATP) from glucose [8, 9]. This hallmark metabolic pathway was first reported by Warburg in 1956 and is known as the Warburg effect [10]. Conversely, it has been suggested that the compensatory aerobic glycolysis in tumor cells is independent from the normal mitochondrial oxidative phosphorylation because it exerts no impact on the fundamental function of the mitochondria [11]. However, the detailed molecule network of glycolysis still needs to be further explored in NSCLC.

Several key enzymes and transcription factors are involved in regulating cellular metabolism in cancer development (e.g., the proto-oncogene *C-myc*) [12, 13]. According to previous studies, C-myc upregulates lactate dehydrogenase-A (LDHA) and hexokinase-2 (HK-2) gene expression [13, 14]. These two genes encode the crucial enzymes LDHA and HK-2, respectively, required for the catalytic reactions in glycolysis—the former enzyme converts pyruvate into lactate, whereas the latter phosphorylates glucose [15, 16].

Proteins containing the RING finger domain can mediate this ubiquitination, and they actively regulate the target protein [17–19], and participating in the regulation of tumorigenesis, particularly in NSCLC, and serving as the prospective biomarkers for cancer clinical management [20, 21]. RING finger domain RING finger protein 180 (RNF180) acts as a tumor suppressor in gastric cancer [22]. Moreover, recent publication has demonstrated that RNF180 is associated with biological behavior and prognosis in patients with NSCLC [23]. However, the precise function of RNF180 and molecule mechanism in NSCLC remain unclear.

In the present study, we investigated the function and molecule network of RNF180 in the proliferation and glycolysis in human NSCLC cells. Our findings demonstrated the correlation between RNF180 expression and mortality in patients with NSCLC, its suppressive effects on NSCLC cell proliferation and glycolytic function, and the underlying mechanisms that involve C-myc downregulation via enhancing its ubiquitination in NSCLC cells.

## Methods

### Human NSCLC tissue samples

In the present study, we have compared the mRNA levels of RNF180 by using 30 paired tumorous. Moreover, a total of 93 NSCLC tissues that excluded patients receiving chemotherapy or radiotherapy were used for immunohistochemical (IHC) assay. All patients provided written informed consent. This study was approved by the independent ethics committee of the First Affiliated Hospital of Soochow University and was performed in accordance with the Declaration of Helsinki.

### Cell lines and cultures

Human NSCLC A549, H292, H358, H1975, and PC9 cells were purchased from ATCC (Manassas, VA, USA) and cultured in Hyclone DMEM/F12 (SH30023.01B) that contains 10% fetal bovine serum 16000-044 (Gibco, USA) supplementation and 100 U/mL of penicillin (Solarbio, China). Cells were grown as adherent cultures under 5% CO_2_ at 37°C in a cell incubator.

### RNF180 overexpression vector construction and transfection

The primers for human RNF180 gene (NM_152925.2) were as follows: 5′-CGGAATTCATGAAAAGAAGCAAAGAATTGATAAC-3′ (EcoR I) (forward) and 5′-CGGGATCCCTAAAACGGAAAGAAAAAATAGC-3′ (BamH I) (reverse). The cDNA fragment encoding RNF180 was inserted into pLVX-Puro (Clontech) between the cloning sites EcoR I and BamH I (underlined). Plasmids pLVX-Puro-RNF180, pMD2G, and psPAX2 (Addgen, USA) were packaged into lentiviruses via 293T cells using Lipofectamine™ 2000 (Invitrogen, USA). After 4–6 h, the cells were transferred into a complete medium, following which the lentiviruses were harvested after 48 and 72 h. Thereafter, lentiviruses (~1.5 μg) possessing RNF180 expression were transfected into the 293T cells; the transfected cells were considered the oeRNF180 group. The cells transfected with lentiviruses with no RNF180 expression were considered the vector group for control.

### RNF180 silencing vector construction and transfection

The lentivirus vectors comprised PLKO.1, pMD2G, and psPAX2. A short-hairpin RNA (shRNA) whose expression was controlled using a U6 promoter was contained in the PLKO.1 vector. A 20–23-nt gene fragment from the RNF180 cDNA was selected as the siRNA target (siRNA-RNF180). The sequences of siRNA-RNF180 were as follows: siRNF180-1 (Site: 219-237), GGAGTATCTTGAGAATCAA; siRNF180-2 (Site: 1211-1229), GCATTAATCAGAGGCTTAA; and siRNF180-3 (Site: 1748-1766), GGATGGATTACCTGCACTT.

The target siRNA-RNF180 was introduced into the PLKO.1 (pLKO.1-shRNF180), and the accuracy of insertion was analyzed (Shanghai Majorbio Bio-Pharm Technology Co., Ltd, China). The lentivirus vectors were constructed followed by co-transfection in the 293T cells according to the abovementioned methods. The control shRNA vector was similarly constructed and transfected. NSCLC (H292) cells containing RNF180-siRNA-pLKO.1 were considered the siRNA-RNF180 group; cells containing the control shRNA vector were used as the control group (siNC).

### Xenograft model

In this section, 20 nude mice (aged 4–6 weeks) were purchased from Shanghai Laboratory Animal Company (Shanghai, China) and randomly allocated into the oeNC and oeRNF180 groups. Mice in both groups were subcutaneously injected with 7 × 10^5^ oeNC- and oeRNF180-transfected H358 cells. In each group, mice were reared with independent feeding and regular bedding changes. Tumor volume (mm^3^) was measured every third day from day 12 to day 33 and was calculated as follows: length × (width^2^/2). On day 33, the mice were sacrificed. Further, the tumor tissues were weighed (g).

The in vivo study was approved by the ethics committee of the First Affiliated Hospital of Soochow University. All mice were handled according to the Institutional Animal Care and Use Committee guidelines, and the experiments were conducted following its guidelines for animal experimentation.

### Cell proliferation assay

Proliferation of NSCLC cells was assessed using the Cell Counting Kit-8 (CCK-8). The control or treated cells in individual wells were mixed with 10 mL of CCK-8 reagent and 90 mL of the 0, 24, 48, or 72h incubated serum-free culture medium, followed by another 1h incubation. A microplate reader (Bio-Rad, USA) was used for measurement at 450-nm optical density.

### Immunohistochemical assay

The tumorous or normal lung tissue sections (4–7 μm in thickness) were incubated with RNF180 antibodies (ab127548, Abcam, UK) overnight at 4°C, followed by incubation with secondary antibodies (D-3004; Long Island Biotech, China) for 30 min at 25°C. For IHC staining, 3,3′-diaminobenzidine (DAB) substrate (Long Island Biotech, USA) and hematoxylin 714094 (BASO Diagnostic Inc., China) were used. The ECLIPSE Ni-E/Ni-U microscope (Nikon, Japan) with DS-Ri2 imaging system (Nikon) was used to visualize RNF180-positive cells. The tumor cells with a positive stain of >25% were considered as having high RNF180 expression according to the methods as previous reports mentioned [24, 25], whereas those with <25% were considered as having low RNF180 expression.

### Western blotting

The level of total protein in the supernatant of the NSCLC cell lysis was determined by the BCA (bicinchoninic acid) Protein Assay Kit (Thermo Fisher Scientific, China). The samples were boiled for 10 min at 95°C. An aliquot of 30 mg of protein was separated using 10% SDS–PAGE gel, followed by PVDF membrane transfer, blocking for 1 h in 5% skim milk, and incubation with separate antibodies (Abcam, Inc., USA)—1:1000 diluted antibody Ab127548 (anti-RNF180), 1:500 diluted antibody Ab39688 (anti-c-Myc), 1:5000 diluted antibody Ab227198 (anti-HK-2), 1:1000 diluted Ab101562 antibody (anti-LDHA), and 1:2000 diluted anti-GAPDH antibody (#5174, Cell Signaling Technology)—overnight at 4°C. Thereafter, the membranes were incubated with horseradish peroxidase secondary antibodies (A0208 (1:200), A0181(1:200), and A0216(1:200); Beyotime Biotechnology) at room temperature for 1 h. The ECL plus substrate (GE Healthcare, USA) with the LAS-400 Image Analyzer (FujiFilm Medical Systems, USA) was used for the detection of the horseradish peroxidase signal.

### Quantitative real-time polymerase chain reaction (qRT-PCR)

TRIzol reagent (Invitrogen, USA) was used for total RNA extraction from cultured cells or tissue samples. The first-strand cDNA synthesis was performed using the RevertAid First Stand cDNA Synthesis Kit (Fermentas, USA). Quantified analysis of RNF180 and GAPDH mRNA levels was conducted using the SYBR Green Master Mixes (Thermo Fisher Scientific, China) on 7300 Real-Time PCR System (Applied Biosystems, USA). GAPDH was used for normalization. The sequences of the related primers used were as follows: RNF180, F 5′-TGACTTTCCTGATGGACCTG-3′, R 5′-ATCCCACTCCTGAGTATTTACC-3′; C-myc, F 5′-TCCTGTCCGTCCAAGCAG-3′, R 5′-ACGCACAAGAGTTCCGTAG-3′ and GAPDH, F 5′-GGATTGTCTGGCAGTAGCC-3′, R 5′-ATTGTGAAAGGCAGGGAG-3′.

### Analysis of cellular aerobic glycolysis and mitochondrial respiration

The extracellular acidification rate (ECAR) of the H358 cells was measured and analyzed using the Seahorse XFe24 Analyzer (Seahorse Bioscience, USA). Approximately 50,000 target cells per well in a 24-well plate (Seahorse Bioscience) with overall 250 μL of culture medium were seeded, followed by overnight incubation with 5% CO_2_ at 37°C.

For the ECAR assay, the adherent cells were washed with phosphate-buffered saline and resuspended in 500 μL of XF Base Medium (pH 7.4; Seahorse Bioscience) containing 2 mM of L-glutamine (basal conditions), 25 mM of glucose (main substrate in aerobic glycolysis), 1 μM of oligomycin (oxidative phosphorylation inhibitor), or 50 mM of 2-deoxy-D-glucose (2-DG) (glycolysis inhibitor) in real-time conditions. For the OCR assay, the adherent cells were maintained in XF Base Medium containing 1 μM of oligomycin, 1 μM of protonophore trifluoromethoxy carbonyl cyanide phenylhydrazone (FCCP), and 0.5 μM of antimycin A/rotenone in real-time conditions. The applications of oligomycin (mitochondrial inhibitors) for ATP synthase blocking, FCCP for inner mitochondrial membrane permeability induction, and rotenone and antimycin A for Complex I and III inhibition, respectively, were required for both assays.

### Immunofluorescence

Cells were mounted onto microscope slides with a 30-min fixation using 4% formaldehyde, followed by 10-min permeabilization using 0.5% Triton X-100 (Solarbio, China). After 30-min blocking using 1% bovine serum albumin (Solarbio, China), the cells were incubated with antibody ab15580 (anti-Ki67, Abcam) overnight at 4°C; thereafter, they were further incubated in the dark with a secondary antibody (Beyotime Biotechnology, China) for 30 min at 37°C. For the cell nuclei staining, 2-(4-amidinophenyl)-6-indolecarbamidine dihydrochloride (DAPI) from Beyotime Biotechnology was used. The ECLIPSE Ni Fluorescent microscope (Nikon) was used for visualization.

### Co-immunoprecipitation and in vitro ubiquitination assay

Approximately 100 μg of the immune complexes in the lysed NSCLC cell supernatant were harvested using Protein A/G PLUS-Agarose (Santa Cruz Biotechnology, USA). IgG sc-2027 from Santa Cruz Biotechnology, anti-RNF180 antibody Orb2721 from Biorbyt, and anti-c-Myc antibody ab32072 from Abcam were used in the co-immunoprecipitation (Co-IP) assays. Anti-RNF180 and anti-c-Myc antibodies (ab127548 and ab39688) from Abcam were used in western blotting. The quantity of total protein was controlled. The anti-ubiquitin antibody ab7780 (Abcam) was used to determine the c-Myc ubiquitination in the precipitation of the immune complexes.

### Statistical analyses

Data were presented as mean ± SEM, which were calculated from three parallels in each experiment. Comparison between groups was conducted using one-way analysis of variance and post-hoc Tukey’s test. Statistical significance was determined at *P* value of <0.05.

## Results

### RNF180 expression was downregulated in NSCLC cells

**Fig. 1:**
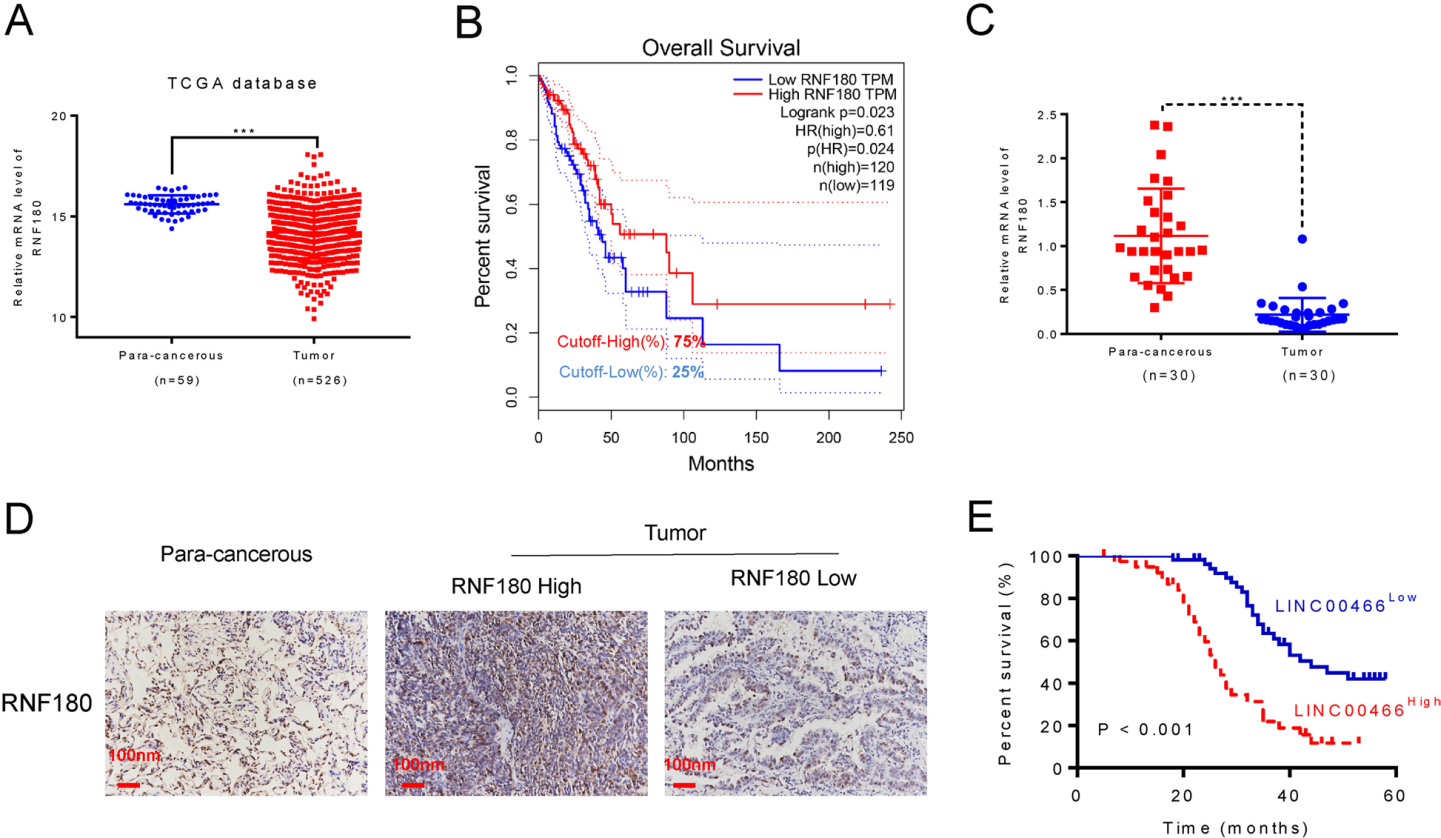
RNF180 was downregulated in non-small cell lung cancer (NSCLC) and positively correlated with its overall survival rate. **A** RNF180 expression in patients with lung adenocarcinoma (LUAD) (*n* = 515) and healthy individuals (*n* = 59) was analyzed based on the TCGA database. *** *p*<0.001 vs para-cancerous. **B** Survival probability of patients with LUAD exhibiting high RNF180 expression (*n* = 126) and low-to-medium RNF180 expression (*n* = 376) was analyzed using the Kaplan–Meier method. **C** RNF180 mRNA levels in 30 pairs of tumorous and adjacent normal tissues were measured using qRT-PCR. *** *p*<0.001 vs para-cancerous. **D** Representative IHC image of RNF180 expression in tissues of healthy controls (*n* = 5) and patients with NSCLC (*n* = 93) was assessed using the IHC assay (original magnification 200×). Representative IHC image is presented. **E** Overall survival probability of patients with NSCLC was analyzed using the Kaplan–Meier method and compared between high RNF180-expressed (*n* = 53) and low RNF180-expressed (*n* = 40) groups

To analyze the expression profile of RNF180 in NSCLC, we collected the data from the TCGA database (http://ualcan.path.uab.edu/analysis.html). As shown in Fig. 1A, the levels of RNF180 were significantly downregulated in patients with NSCLC (*n*=526) compared with that in pare-cancerous tissues (*n*=59). Moreover, it was easily identified that low expression of RNF180 was associated with the poor survival rate in NSCLC patients (Fig. 1B). Furthermore, a total of 30 pairs of tumorous and adjacent para-cancerous tissues were used to determine the relative mRNA levels of RNF180. Importantly, our results suggested that the expression of RNF180 was significantly lower in patients with NSCLC compared with that in normal individuals (Fig. 1C). Next, IHC assay was used to examine the protein expression of RNF180 in tumor tissues and para-cancerous. In 93 patients, we observed 53 tumorous tissues exhibiting high level of RNF180 expression, where the number of 40 tissues with were identified with low level of RNF180 expression (Fig. 1D). Using the Kaplan–Meier method, a significantly lower survival rate was observed in patients NSCLC with high RNF180 expression compared with that observed in patients with low RNF180 expression throughout the 60-month experiment duration (Fig. 1E).

### RNF180 overexpression inhibited the proliferation and glycolysis activity of human H292 cells

To further examine the function of RNF180, we have examined the expression of RNF180 in five NSCLC cell lines, including A549, H358, H292, H358, and PC9. The normal human lung bronchial epithelial (HBE) was used as control. Clearly, the relative mRNA and protein levels RNF180 were significantly downregulated in human NSCLC cell lines, especially in H1975 and H358 (Supplementary Figure S1A-S1B). Then, RNF180 was successfully induced overexpression in H1975 and H358 using lentiviral-mediate vector (Supplementary Figure S1C-S1D). Moreover, the RNF180 shRNAs (shRNF180-1, shRNF180-2 and shRNF180-3) were significantly suppressed the endogenous expression of RNF180 in H292 cells (Supplementary Figure S1E-S1F).

**Fig. 2:**
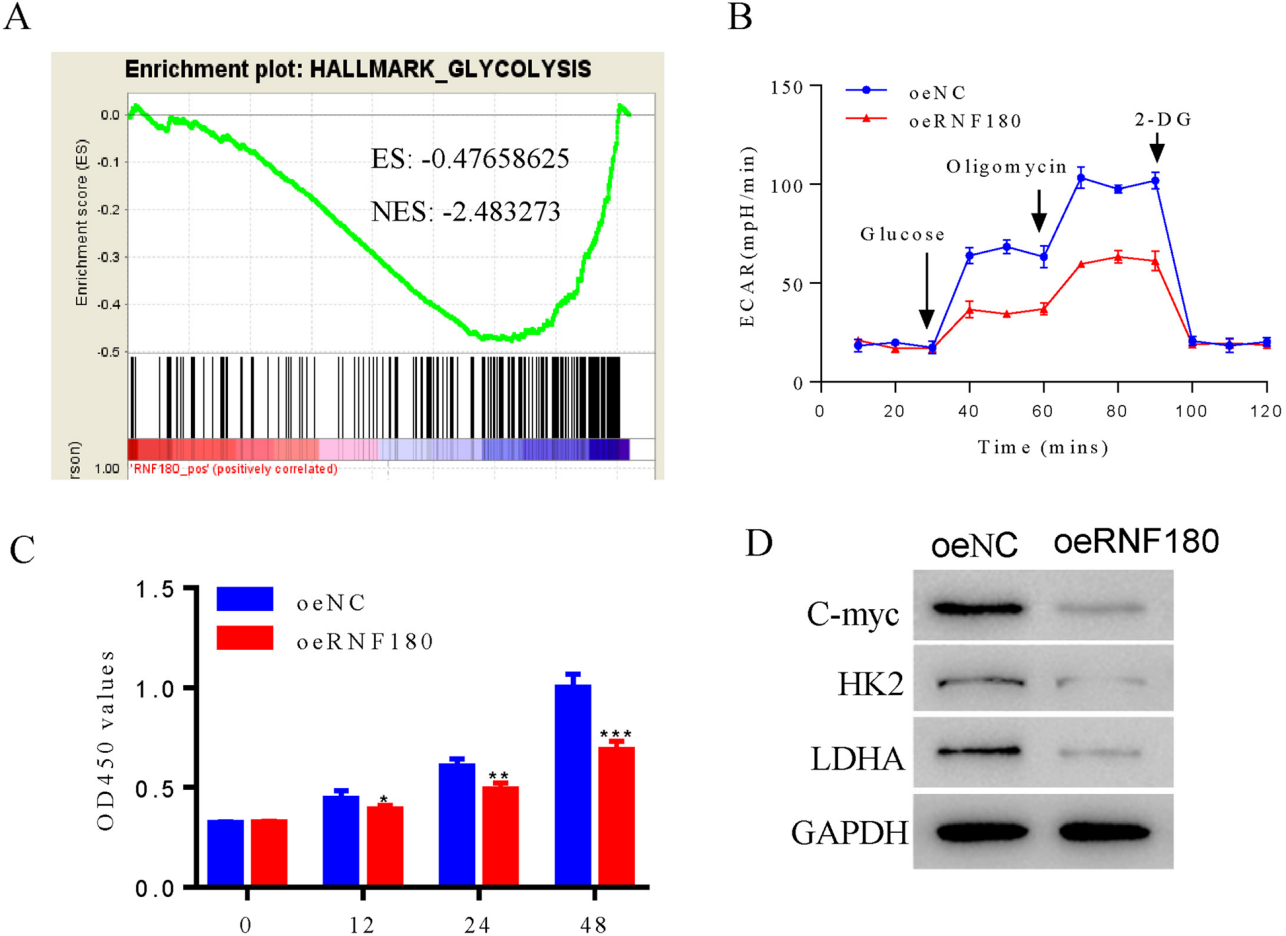
Overexpression of RNF180 decreased the activity of glycolysis and proliferation of human H292 cells. **A** Functional analysis indicated that RNF180 was enriched in glycolysis metabolism in NSCLC. **B** Overexpression of RNF180 significantly suppressed the level of ECAR in NSCLC cells. **C** CCK8 assay was performed to examine the proliferation of oeNC and oeRNF180 transfecting cells respectively. **D** Western blot was used to examine the protein levels of C-myc, HK2, and LDHA in oeNC and oeRNF180 transfecting cells

Based on TCGA database and gene set enrichment analysis, RNF180 was negatively correlated with glycolysis metabolism (Fig. 2A). In the present study, ECAR assay was used to determine the function of RNF180 in the glycolysis of NSCLC cells. As shown in Fig. 2B, overexpression of RNF180 significantly suppressed the glycolysis activity of human H292 cells. Moreover, our results indicated that oeRNF180 significantly suppressed the proliferation of human H292 cells (Fig. 2C). Interestingly, the protein levels of C-myc, HK-2, and LDHA were significantly decreased in human NSCLC cells after transfecting with oeRNF180 (Fig. 2D).

### RNF180 overexpression inhibited the tumorigenicity of human H292 cells in vivo

**Fig. 3:**
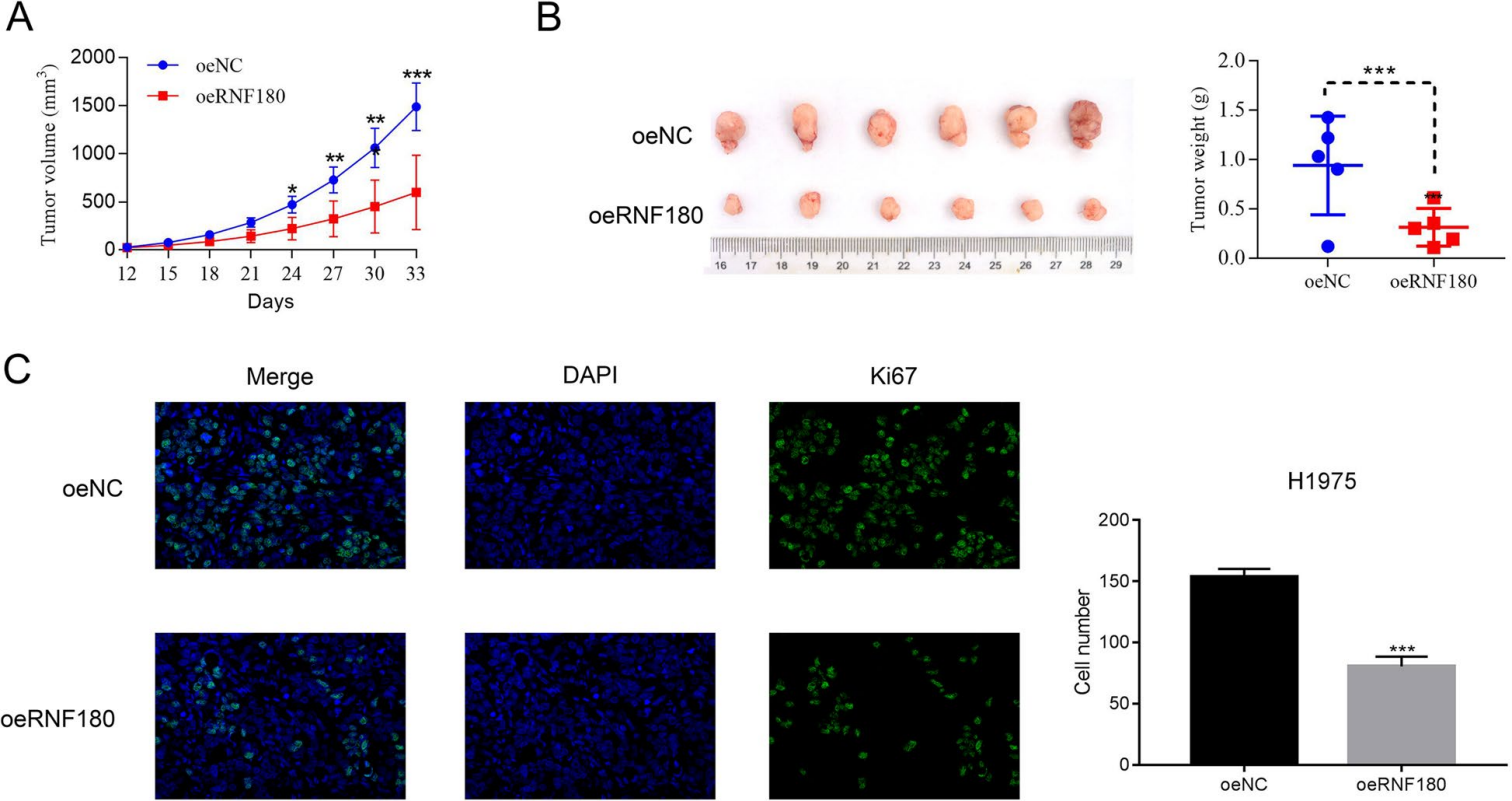
Overexpression of RNF180 inhibited the tumorigenicity of human H292 cells in vivo. **A**, **B** The tumor volume and weight were significantly downregulated in oeRNF180-transfecting cells *in vivo*. * *p* < 0.05 vs oeNC, ** *p* < 0.01 vs oeNC, *** *p* < 0.001 vs oeNC. **C** Immunofluorescence staining for the DAPI-positive (blue) and Ki67-positive (green) cells is shown. ^***^ *p* < 0.01 vs oeNC

To investigate the in vivo effect of RNF180 overexpression on NSCLC tumorigenicity, we subcutaneously injected the oeNC or oeRNF180-transfected H292 cells into nude mice (*n*=5). As shown in Fig. 3A, B, both the tumor volume and weight in oeRNF180 tumor were significantly lower than those in oeNC tumor.

Moreover, Ki67 immunofluorescence staining assay was performed to examine the proliferation of oeNC or oeRNF180 tumor. Clearly, the positive Ki67 cells were much lower in oeRNF180 transfecting cells. Hence, our results suggested that overexpression of RNF180 inhibited the proliferation of H292 cells in vivo (Fig. 3C).

### RNF180 interacted with c-Myc and enhanced its ubiquitination in NSCLC cells

**Fig. 4:**
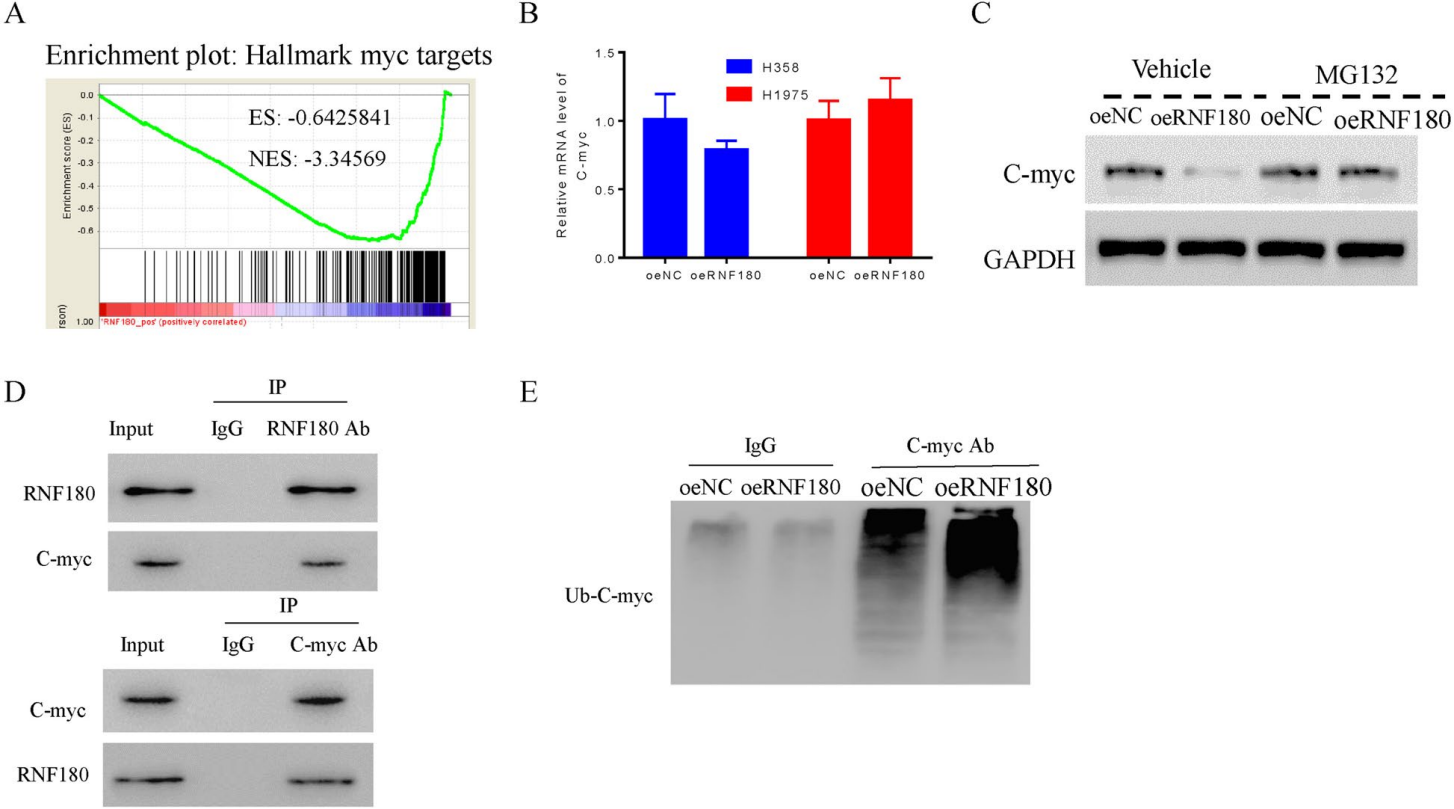
RNF180 interacted with c-Myc and enhanced its ubiquitination in NSCLC cells. **A** Functional analysis indicated that RNF180 negatively correlated with C-myc. **B** qRT-PCR was used to examine the relative mRNA levels of C-myc in oeNC and oeRNF180 transfecting H358 and H1975 cells. **C** Western blot was used to examine the protein levels of C-myc in oeNC or oeRNF180 transfecting cells with or without the treatment of the proteasome inhibitor MG132. **D** CO-IP assay indicated that RNF180 interacted with C-myc. **E** Overexpression of RNF180 enhanced the ubiquitination of C-myc in NSCLC cells

In the present study, bio-information analysis indicated that C-myc is negatively correlated with RNF180 in NSCLC cells (Fig. 4A). To further determine the relationship between RNF180 and C-myc, qRT-PCR was used to determine the mRNA levels of C-myc in oeNC and oeRNF180 transfecting cells. As shown in Fig. 4B, the relative mRNA level of C-myc showed no significance difference between oeNC and oeRNF180 transfecting cells, while significantly inhibited its protein expression. Moreover, the proteasome inhibitor MG132 largely abolished the function of oeRNF180 in NSCLC cells (Fig. 4B, C). Moreover, results obtained from CO-IP assay suggested that RNF180 interacted with C-myc (Fig. 4D). Overexpression of RNF180 significantly promoted the ubiquitination of C-myc in NSCLC cells (Fig. 4E). Importantly, the similar results were also obtained in RNF180 shRNA transfecting cells (Figure S2).

### c-Myc inhibition counteracted the effects of shRNF180 on NSCLC cell proliferation and glycolysis metabolism

**Fig. 5:**
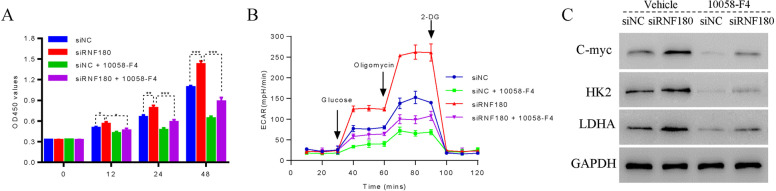
C-myc inhibition counteracted the effects of shRNF180 on NSCLC cell proliferation and glycolysis metabolism. **A** The proliferation of siRNF180 transfecting cells was inhibited after co-cultured with the C-myc inhibitor 10058-F4. * *p* < 0.05, ** *p* < 0.01, and *** *p* < 0.001. **B** The inhibitor 10058-F4 inhibited the levels of ECAR in siRNF180 transfecting cells. **C** Western blot was used to examine the protein levels of C-myc, HK2, and LDHA in cells as indicated above

To further examine the connection between C-myc and RNF180, the C-myc inhibitor 10058-F4 was used to culture shRNF180 transfecting cells. As shown in Fig. 5A, knockdown of RNF180 promoted the proliferation of NSCLC cells, while was downregulated after co-culture with the inhibitor 10058-F4. Moreover, the level of ECAR of shRNF180 transfecting cells was highly increased. Importantly, the inhibitor 10058-F4 significantly suppressed the function of shRNF180 in NSCLC cells (Fig. 5B). Moreover, the relative c-Myc, HK-2, and LDHA protein levels in the siRNF180 cells were significantly elevated compared with those in the siNC cells (Fig. 5C). However, the c-Myc inhibitor 10058-F4 significantly reduced the c-Myc, HK-2, and LDHA levels (*P* < 0.01). Together, 10058-F4 treatment significantly counteracted the stimulation of c-Myc, HK-2, and LDHA in the siRNF180 cells and decreased their expression levels (*P* < 0.01).

## Discussion

NSCLC is a major cause of cancer-related death in human worldwide. However, the moleculer pathogenesis still need to be further explored. It has been identified that Katanin P60 is a potential biomarker for lymph node metastasis and prognosis for non-small cell lung cancer [26]. Moreover, circular RNAs are reported to play an essential role in the progression of human lung cancer [27]. Recently, Wang et al. has discovered that SCLC-P was associated with smokers and is one of the poor prognostic factors of limited-stage small cell lung cancer [28]. Furthermore, AURKB, CCNB2, CDC20, CDCA5, CDCA8, CENPF, and KNTC1 are were highly expressed in lung cancer tissues and were associated with poor prognosis [29]. Lee et al. have illustrated that EpCAM and TROP2 were significantly overexpressed in in non-small cell lung cancer [30]. Furthermore, stem-cells are reported to associate with the progression of renal cell cancer [31]. Therefore, enhancing the comprehension in the mechanism of lung cancer contributed to develop the therapy.

Proteins containing the RING finger domain are mediators of either E2 ubiquitin-conjugating enzyme-dependent or E3 ubiquitin ligase-dependent ubiquitination, essentially participating in regulating multiple biological processes including cellular apoptosis and carcinogenesis [32, 33]. In the present study, our results indicated that RNF180 was found to be significantly reduced in NSCLC and positively correlated with overall survival rate of NSCLC patients. Therefore, these results demonstrated the potential use of RNF180 as a biomarker for NSCLC prognosis.

RNF180 is a membrane-bound E3 ubiquitin ligase [34], and its function as a tumor inhibitor in gastric cancer has previously been studied [35]. Deng et al. (2016) demonstrated that RNF180 acts as a tumor suppressor gene in gastric cancer, and its antitumor effects include inhibiting cell proliferation and repressing tumor growth in vivo, which was further confirmed for NSCLC in the present study.

In addition, the direct and indirect functions of RNF for regulating cellular energy metabolism have previously been documented [36, 37]. Although ECAR has some limitations in determining the activity of aerobic glycolysis, it is broadly used as a good marker in recent research [38]. In the present study, RNF180 was observed to restrict both aerobic glycolysis by using ECAR method and the molecular mechanisms involving the suppression of c-Myc protein levels and its subsequent downstream proteins HK-2 and LDHA related to energy metabolism. These findings identified the inhibiting effect of RNF180 in NSCLC energy metabolism.

The RNF family are reportedly active for interacting with c-Myc proteins [18–20]. Furthermore, we investigated whether C-myc was the mechanism via which RNF180 regulated NSCLC cell proliferation and energy metabolism. Our data confirmed the inhibiting effects of the C-myc inhibitor on NSCLC cell proliferation and glycolytic function. These effects were significantly abolished by RNF180 knockdown. The ubiquitination-dependent degradation of C-myc reportedly counteracts carcinogenesis and tumor progression [39, 40]. The results of the present study suggested the antitumor effects of RNF180 via C-myc downregulation through ubiquitination-dependent degradation. Moreover, our present findings not only enhanced the understanding of RNF180 as an anti-oncogene in the progression of lung cancer but also provided evidences to indicate its potential role and molecule pathway as a potential target in developing the therapy for human lung cancer.

## Conclusion

Our findings demonstrated the inhibitory effects of RNF180 on NSCLC cell proliferation, metabolic activities, and tumorigenicity as well as the potential underlying mechanisms. To date, this is the first study to demonstrate the antitumor function of RNF180 in lung cancer and indicated its potential values as a target in NSCLC therapy.

## Supplementary Information

**Additional file 1: Figure S1**. Knockdown and overexpression of RNF180 in NSCLC cells. A and B. qRT-PCR was used to examine the relative mRNA and protein levels of RNF180 in human NSCLC cell lines, including A549, H1975, H292, H358 and PC9. The normal human lung bronchial epithelial (HBE) was used as control. *** *p* < 0.001 vs oeNC. C and D. Lentiviral- mediate vector was used to induce RNF180 overexpression in H1975 and H358 cells respectively. *** *p* < 0.001 vs oeNC. E and F. RNF180 shRNAs (shRNF180-1, shRNF180-2 and shRNF180-3) were used to silence the expression of RNF180 in H292 cells.

**Additional file 2: Figure S2**. RNF180 silencing contributed to the expression of C-myc through inhibiting its ubiquitination. A and B. Western blot was used to examine the protein level of C-myc in siNC and siRNF180 transfecting H292 and A549 with or without the treatment of MG132. C and D. Knockdown of siRNF180 inhibited the ubiquitination of C-myc in H292 and A549 cells respectively.

## Data Availability

The datasets used and/or analyzed during the current study are available from the corresponding author on reasonable request.

## Abbreviations

NSCLC: n=Non-small cell lung cancer
RNF180: RING finger protein 180
ATP: Adenosine triphosphate
qRT-PCR: Quantitative real-time polymerase chain reaction
CCK-8: Cell Counting Kit-8
LDHA: Lactate dehydrogenase-A
HK2: Hexokinase-2
IHC: Immunohistochemistry
shRNA: Short-hairpin RNA
OCR: Oxygen consumption rate
ECAR: Extracellular acidification rate
DAPI: 2-(4-Amidinophenyl)-6-indolecarbamidine dihydrochloride
FCCP: Trifluoromethoxy carbonyl cyanide phenylhydrazone
Co-IP: Co-immunoprecipitation
HBE: Human lung bronchial epithelial
