# Correction: Ring finger protein 180 suppresses cell proliferation and energy metabolism of non-small cell lung cancer through downregulating C-myc

**DOI:** 10.1186/s12957-022-02690-3

**Published:** 2022-07-14

**Authors:** Yi Ding, Yi Lu, Xinjie Xie, Lei Cao, Shiying Zheng

**Affiliations:** 1 Department of Thoracic Surgery, The First Afliated Hospital of Soochow University, NO. 188, Shizi Street, Suzhou, 215006 People’s Republic of China; 2 Department of Thoracic Surgery, Shanghai Pudong New Area People’s Hospital, Shanghai University of Medicine and Health Sciences, Shanghai, 201318 People’s Republic of China; 3 Department of Pathology, Shanghai Pudong New Area People’s Hospital, Shanghai University of Medicine and Health Sciences, Shanghai, 201318 People’s Republic of China

**Correction: World J Surg Oncol 20, 162 (2022)**

**https://doi.org/10.1186/s12957-022-02599-x**

Following the publication of the original article, the author reported an error in Fig. 1E. The correct figure is included in this correction and the original article has been updated.

**Fig. 1:**
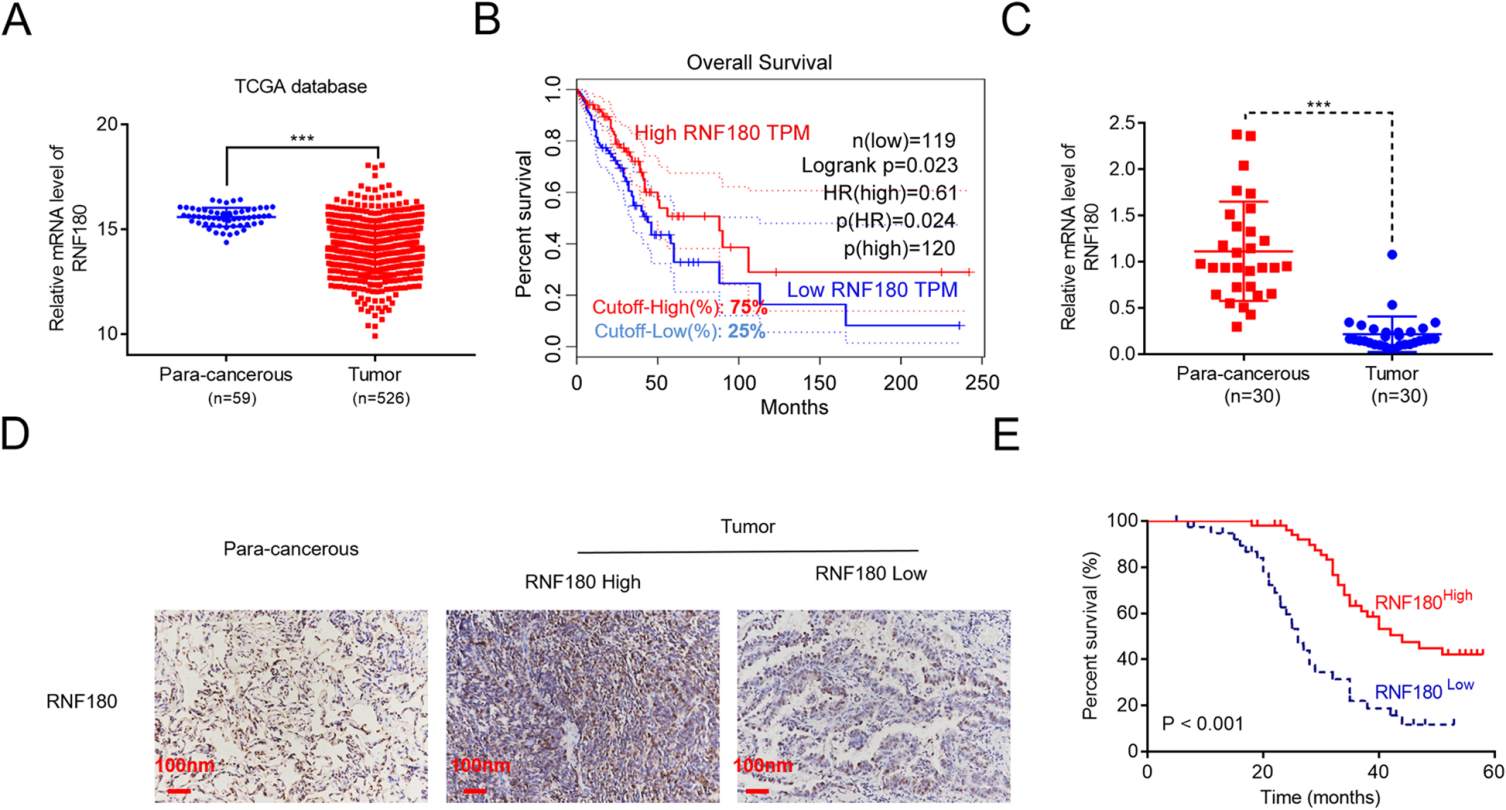
RNF180 was downregulated in non-small cell lung cancer (NSCLC) and positively correlated with its overall survival rate. **A** RNF180 expression in patients with lung adenocarcinoma (LUAD) (*n* = 526) and healthy individuals (*n* = 59) was analyzed based on the TCGA database. *** *p*<0.001 vs para-cancerous. **B** Survival probability of patients with LUAD exhibiting high RNF180 expression (*n* = 120) and low-to-medium RNF180 expression (*n* = 119) was analyzed using the Kaplan–Meier method. **C** RNF180 mRNA levels in 30 pairs of tumorous and adjacent normal tissues were measured using qRT-PCR. *** *p*<0.001 vs para-cancerous. **D** Representative IHC image of RNF180 expression in tissues of healthy controls (*n* = 5) and patients with NSCLC (*n* = 93) was assessed using the IHC assay (original magnification 200×). Representative IHC image is presented. **E** Overall survival probability of patients with NSCLC was analyzed using the Kaplan–Meier method and compared between high RNF180-expressed (*n* = 53) and low RNF180-expressed (*n* = 40) groups
