# Supplementary figures and images for: Ring finger protein 180 suppresses cell proliferation and energy metabolism of non-small cell lung cancer through downregulating C-myc
Source: World J Surg Oncol. 2022 May 21;20:162. doi: 10.1186/s12957-022-02599-x (PMC9123707; doi:10.1186/s12957-022-02599-x)

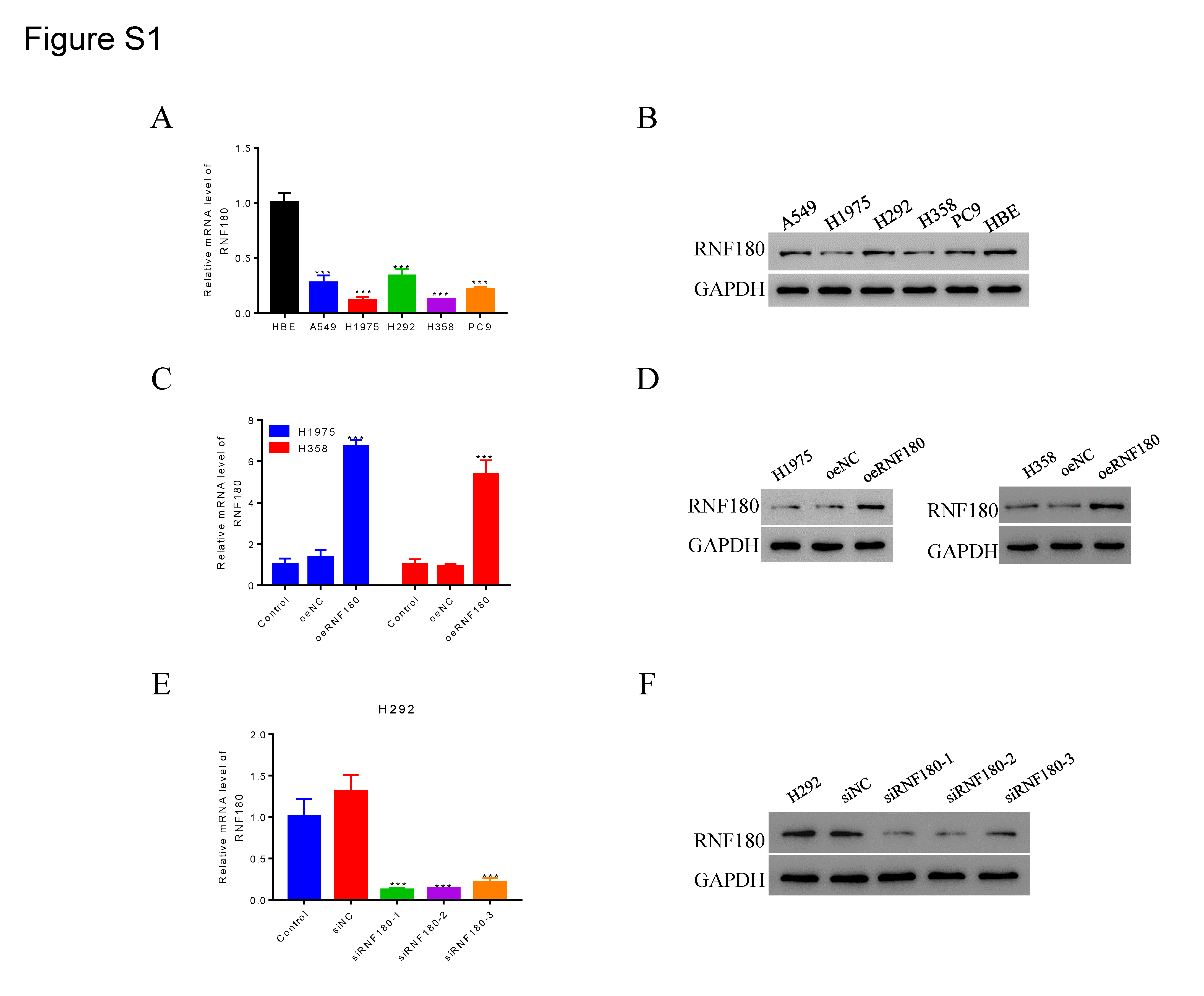

Supplement: Supplementary file 1 — Additional file 1: Figure S1. Knockdown and overexpression of RNF180 in NSCLC cells. A and B. qRT-PCR was used to examine the relative mRNA and protein levels of RNF180 in human NSCLC cell lines, including A549, H1975, H292, H358 and PC9. The normal human lung bronchial epithelial (HBE) was used as control. *** p < 0.001 vs oeNC. C and D. Lentiviral- mediate vector was used to induce RNF180 overexpression in H1975 and H358 cells respectively. *** p < 0.001 vs oeNC. E and F. RNF180 shRNAs (shRNF180-1, shRNF180-2 and shRNF180-3) were used to silence the expression of RNF180 in H292 cells. [file 12957_2022_2599_MOESM1_ESM.jpg]

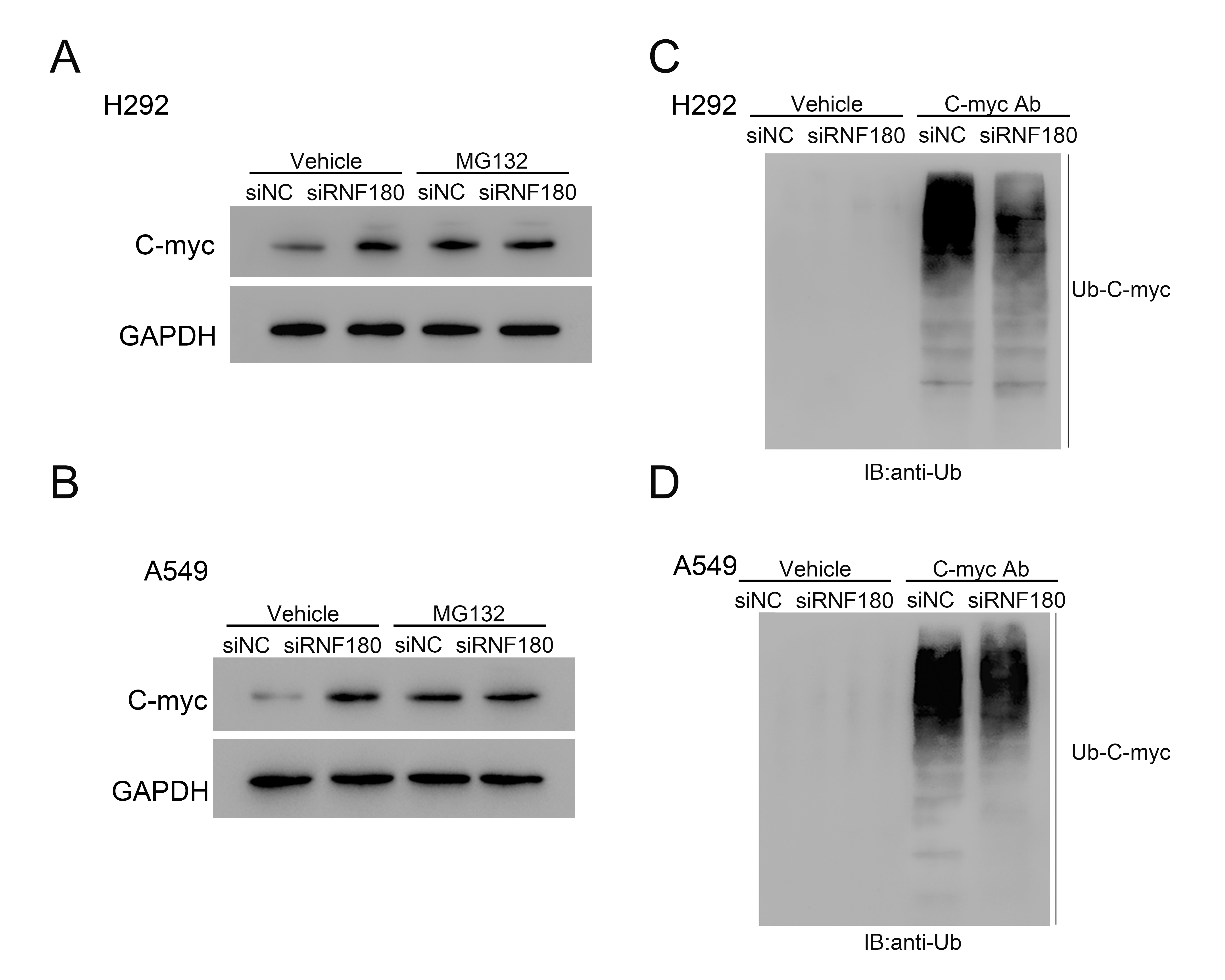

Supplement: Supplementary file 2 — Additional file 2: Figure S2. RNF180 silencing contributed to the expression of C-myc through inhibiting its ubiquitination. A and B. Western blot was used to examine the protein level of C-myc in siNC and siRNF180 transfecting H292 and A549 with or without the treatment of MG132. C and D. Knockdown of siRNF180 inhibited the ubiquitination of C-myc in H292 and A549 cells respectively. [file 12957_2022_2599_MOESM2_ESM.jpg]
